# Positive-unlabelled learning of glycosylation sites in the human proteome

**DOI:** 10.1186/s12859-019-2700-1

**Published:** 2019-03-06

**Authors:** Fuyi Li, Yang Zhang, Anthony W. Purcell, Geoffrey I. Webb, Kuo-Chen Chou, Trevor Lithgow, Chen Li, Jiangning Song

**Affiliations:** 1 Infection and Immunity Program, Biomedicine Discovery Institute and Department of Biochemistry and Molecular Biology, Monash University, Melbourne, VIC 3800 Australia; 2 Monash Centre for Data Science, Faculty of Information Technology, Monash University, Melbourne, VIC 3800 Australia; 3 College of Information Engineering, Northwest A and F University, Yangling, 712100 Shaanxi China; 4 Gordon Life Science Institute, Boston, MA 02478 USA; 5 Center for Informational Biology, School of Life Science and Technology, University of Electronic Science and Technology of China, Chengdu, 610054 China; 6 Infection and Immunity Program, Biomedicine Discovery Institute and Department of Microbiology, Monash University, Melbourne, VIC 3800 Australia; 7 Department of Biology, Institute of Molecular Systems Biology, ETH Zürich, 8093 Zürich, Switzerland

**Keywords:** Protein glycosylation prediction, Positive unlabelled-learning, Supervised-learning, AlphaMax, Sequence analysis, Sequence-derived features

## Abstract

**Background:**

As an important type of post-translational modification (PTM), protein glycosylation plays a crucial role in protein stability and protein function. The abundance and ubiquity of protein glycosylation across three domains of life involving Eukarya, Bacteria and Archaea demonstrate its roles in regulating a variety of signalling and metabolic pathways. Mutations on and in the proximity of glycosylation sites are highly associated with human diseases. Accordingly, accurate prediction of glycosylation can complement laboratory-based methods and greatly benefit experimental efforts for characterization and understanding of functional roles of glycosylation. For this purpose, a number of supervised-learning approaches have been proposed to identify glycosylation sites, demonstrating a promising predictive performance. To train a conventional supervised-learning model, both reliable positive and negative samples are required. However, in practice, a large portion of negative samples (i.e. non-glycosylation sites) are mislabelled due to the limitation of current experimental technologies. Moreover, supervised algorithms often fail to take advantage of large volumes of unlabelled data, which can aid in model learning in conjunction with positive samples (i.e. experimentally verified glycosylation sites).

**Results:**

In this study, we propose a positive unlabelled (PU) learning-based method, PA2DE (V2.0), based on the AlphaMax algorithm for protein glycosylation site prediction. The predictive performance of this proposed method was evaluated by a range of glycosylation data collected over a ten-year period based on an interval of three years. Experiments using both benchmarking and independent tests show that our method outperformed the representative supervised-learning algorithms (including support vector machines and random forests) and one-class learners, as well as currently available prediction methods in terms of F1 score, accuracy and AUC measures. In addition, we developed an online web server as an implementation of the optimized model (available at http://glycomine.erc.monash.edu/Lab/GlycoMine_PU/) to facilitate community-wide efforts for accurate prediction of protein glycosylation sites.

**Conclusion:**

The proposed PU learning approach achieved a competitive predictive performance compared with currently available methods. This PU learning schema may also be effectively employed and applied to address the prediction problems of other important types of protein PTM site and functional sites.

## Background

Glycosylation is among the most ubiquitous and important type of post-translational modification (PTM) across three domains of life, including Eukarya, Bacteria, and Archaea [1]. It is estimated that glycosylation may occur in > 50% of the human proteins [2], and that it is ubiquitous in all living organisms [3]. Glycosylation involves attachment of different types of glycan molecules to a specific amino acid side-chain (i.e., tryptophan, asparagine, serine, or threonine) in protein substrates [4]. Glycosylation has been reported to be relevant for a myriad of biological processes, including cell signalling and communication, cell dissociation, immune modulation, protein quality control, protein folding, subcellular localization, and degradation [5–12]. Based on its critical role in a wide variety of major pathways, protein glycosylation is associated with a variety of human diseases, including diabetes [13–15], cancers [16–20], and autoimmune diseases [21–23]. In light of these strong associations with human diseases, and in the current era of precision medicine, there is an urgent need to develop computational tools to accurately predict glycosylation sites in order to prioritize potential candidates for experimental validation and elucidate their biological functions.

To shortlist potential glycosylation sites and facilitate advanced experimental validation, a variety of computational methods have been proposed as useful alternative approaches. Such in silico methods for glycosylation site prediction include NetNGlyc [24], NetOGlyc [21], EnsembleGly [25], GPP [26], GlycEP [27], ModPred [28], as well as our previously developed tools GlycoMine [29] and GlycoMine^struct^ [30], etc. These approaches are based on a supervised-learning scheme [e.g. using supervised learning algorithms such as support vector machines (SVMs), random forest (RF), etc] that uses reliably labelled positive (i.e. experimentally verified glycosylation sites) and negative (i.e. non-glycosylation sites) samples to train the prediction model. In terms of negative sample selection, the majority of current approaches, such as NetNGlyc, NetOGlyc, EnsembleGly, GPP, GlycEP, and GlycoMine^Struct^ randomly selected non-glycosylation sites from experimentally verified glycosylated proteins as the negative samples. However, this strategy can be problematic, as previously assigned negative samples (non-glycosylation sites) could be mislabelled due to limitations in experimental conditions and technologies used, potentially resulting in unreliable negative data selection and biased model training. Moreover, most current methods fail to account for the vast amount of unlabelled data, the majority of which have not been annotated with respect to glycosylation. According to a previous study by De Comite et al. [31], positive and unlabelled samples can aid the learning process. Recently, Niu et al. [32] demonstrated theoretically that positive unlabelled (PU)-learning methods performed better than supervised learning on PU scenarios. Another two recent works highlighted that PU learning can yield an equivalent performance to supervised-learning algorithms [33, 34] when using ranking-based performance measures, such as receiver operating characteristic (ROC) curve and the precision-recall curve. Most recently, a powerful bioinformatics tool, MutPred2 [35], has applied the PU learning approaches to address the problem of inferring the molecular and phenotypic impact of amino acid variants, and achieved a favourable performance compared with supervised-learning algorithms. These studies demonstrate that PU learning has a great capacity to achieve at least competitive performance compared to supervised-learning algorithms and thus effectively avoid the labour-intensive data labelling procedure. Motivated by these studies, in this study, we employed a PU-learning scheme to utilize the vast amount of unlabelled data in order to explore the possibility of achieving a competitive performance compared to the traditional supervised-learning approaches with more relaxed requirement for data labelling.

For the current task, our results suggested that the advantages of PU learning relative to traditional supervised-learning techniques can be summarized as follows: 1) PU learning is fast and simple, is able to significantly reduce the effort and time necessary to label samples and can achieve a competitive performance compared to supervised-learning algorithms [36–38]; and 2) PU-learning is particularly amenable to bioinformatics and computational biology settings, where a sizable portion of previously unidentified samples is likely mislabelled.

In this study, we proposed a novel method, PA2DE (V2.0), under the PU learning scenario for glycosylation sites prediction. We also benchmarked several state-of-the-art PU-learning algorithms and compared the performance of our method with these algorithms for glycosylation prediction using time-scaled datasets collected between 2007 and 2016 and sequence-derived features. The predictive performance of the proposed method was extensively benchmarked against state-of-the-art PU-learning algorithms, traditional supervised-learning algorithms (i.e. SVMs and RFs) and one-class classifiers on both benchmark and independent test datasets. The results showed that PA2DE (V2.0) achieved an outstanding predictive performance in terms of F1 score, accuracy (ACC), and the area under the curve (AUC) values. Next, we retrained the classifiers of PA2DE (V2.0) using a more comprehensive dataset and further compared its predictive performance with several state-of-the-art glycosylation site prediction methods. The performance comparison results demonstrated that PA2DE (V2.0) achieved a competitive performance compared with these methods. Finally, we developed an online web server as an implementation of the proposed method to facilitate the community-wide efforts for performing in silico glycosylation site prediction.

## Results

### Overall framework

**Fig. 1:**
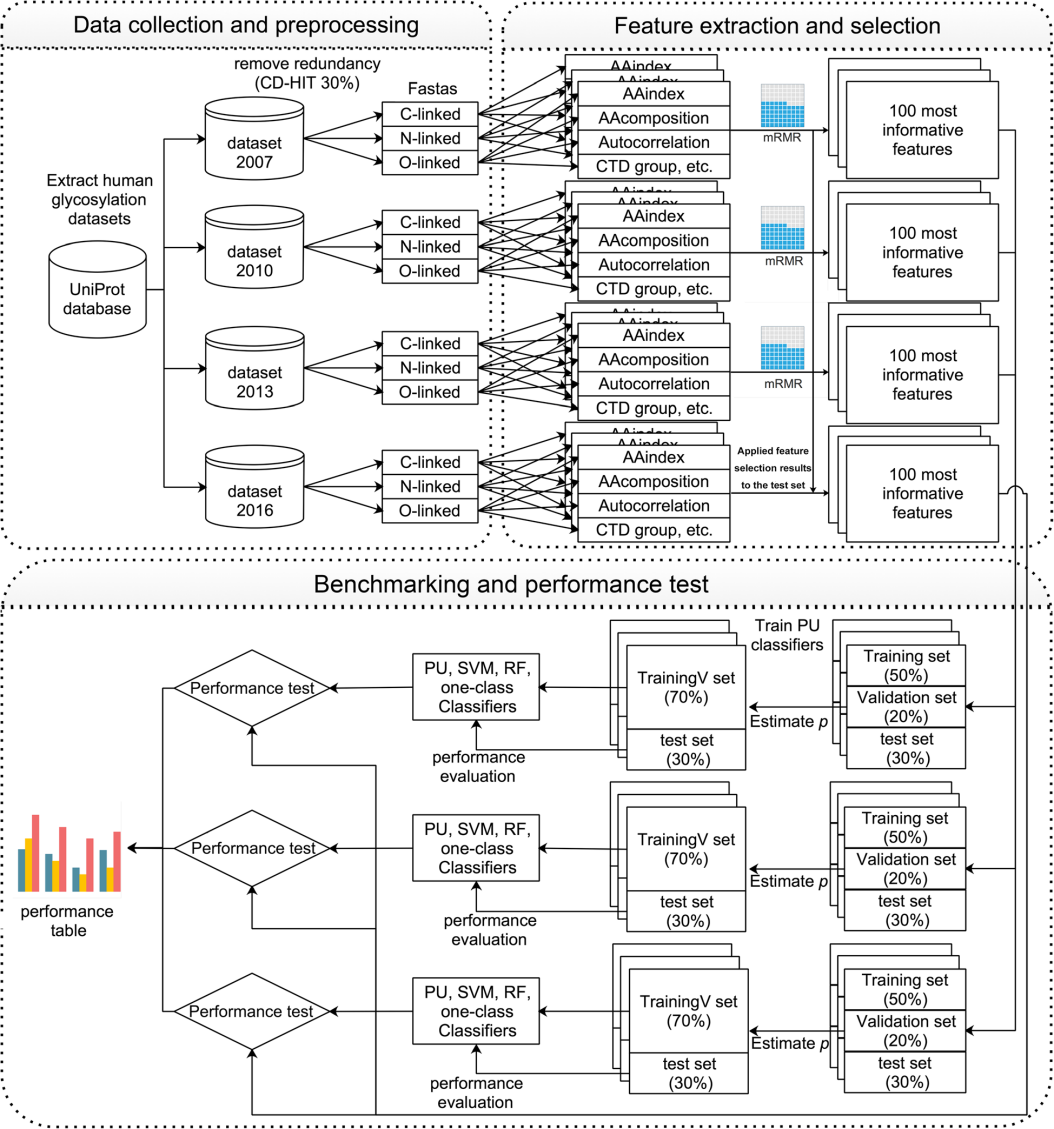
The overall framework of the experiments

Figure 1 illustrates the generic framework used for benchmarking the performance of our proposed method with PU-learning, supervised learning and one-class classification algorithms for glycosylation prediction. As can be shown, this framework comprised three steps, including data collection and pre-processing, feature extraction and selection, and benchmarking and independent tests. At the first step, four time-scaling datasets harbouring experimentally verified C-, N-, and O-linked human glycosylation sites collected in 2007, 2010, 2013, and 2016, respectively, were extracted from the UniProt database [39]. We subsequently performed sequence homology reduction in order to remove the sequence redundancy from the initial datasets. At the second step, a variety of sequence-derived features were calculated and extracted from all four datasets. Feature selection based on the maximal Redundancy Maximal Relevance (mRMR) [40] algorithm was then conducted to eliminate the redundant and irrelevant features. As a result, the top 100 features were ranked and identified for the datasets comprising C-, N-, and O-linked glycosylation data for each year (Refer to the section “Feature extraction and selection”). Please note that the feature selection was only conducted for training sets (i.e. datasets extracted from 2007, 2010, and 2013) and the selection results (i.e. the selected features) were then applied to the test set (i.e. the dataset extracted from 2016). At the final step, we performed the benchmarking and performance tests using these datasets and the correspondingly selected features. Three types of prediction models trained using PU-learning, supervised-learning, and one-class classification algorithms were constructed, evaluated, and compared.

### Dataset analysis

We collected four experimentally verified human C-, N-, and O-linked glycosylation site datasets in the years 2007, 2010, 2013, and 2016 from the UniProt database. A statistical summary of the collected proteins along with their glycosylation sites before and after the sequence-redundancy removal is shown in Table 1.

**Table 1:** A statistical summary of glycosylated proteins and glycosylation sites collected from 2007, 2010, 2013, and 2016 data

Year	Type	Initial dataset prior to redundancy removal		Final dataset after redundancy removal	
		Num. of sites	Num. of substrates	Num. of sites	Num. of substrates
2007	C-linked	36	10	36	10
	N-linked	1245	537	1208	520
	O-linked	321	101	320	100
2010	C-linked	38	12	38	12
	N-linked	2175	908	2118	872
	O-linked	345	114	344	113
2013	C-linked	43	15	43	15
	N-linked	2508	1004	2442	965
	O-linked	474	178	455	162
2016	C-linked	46	17	46	17
	N-linked	2805	1111	2728	1066
	O-linked	698	221	679	212

We first analysed the number of previously mislabelled negative samples (i.e. non-glycosylation sites) in data collected over four years (2007, 2010, 2013, and 2016). The detailed numbers highlighting the previously mislabelled negative sites are shown in Table 2.

**Table 2:** Summary of the results for mislabelled negative sites

Year		C-linked	N-linked	O-linked
2010	N1^a^	0	237 (26.04%)	22 (91.67%)
	P1	11.76%	3.38%	1.26%
	P2	14.86%	3.41%	1.28%
	P3	12.07%	3.39%	1.28%
2013	N1	0	119 (36.73%)	32 (19.82%)
	P1	8.35%	3.01%	1.22%
	P2	9.36%	4.36%	1.24%
	P3	8.62%	3.97%	1.22%
2016	N1	0	99 (34.62%)	32 (19.82%)
	P1	6.51%	3.09%	1.11%
	P2	7.15%	4.68%	1.13%
	P3	6.63%	3.83%	1.13%

Note: a) N1, numbers and percentages of mislabelled non-glycosylation sites and their percentages as compared with previous collection years; b) P1, the actual class probability of glycosylation sites; c) P2: the prior probability of glycosylation sites estimated by the Elkan-Noto algorithm; d) P3: the prior probability of glycosylation sites estimated by the AlphaMax algorithm

In Table 2, the N1 rows of the years 2010, 2013, and 2016 show the numbers of mislabelled non-glycosylation sites and the percentages as compared with those of the corresponding previous collection years (i.e. 2007, 2010, and 2013) for C-, N-, and O-linked glycosylation, respectively. For example, the N1 value of N-linked glycosylation in 2010 was 237, which means that there existed 237 sites, which were labelled as non-glycosylation sites in 2007 but were later labelled as N-linked glycosylation sites in 2010. These 237 mislabelled sites accounted for 26.04% of all newly added sites in 2010 compared with 2007 (e.g. 26.04% = 237/(2118–1208), where 2118 was the number of N-linked glycosylation sites in 2010, while 1208 was the number of N-linked glycosylation sites in 2007). As shown in Table 2, a significant number of non-glycosylation sites were labelled incorrectly due to the limitations of experimental technologies, suggesting the possibility that current non-glycosylation sites might actually represent true positives. With the advances of new technologies, additional previously labelled non-glycosylation sites will likely also become true positives. Importantly, this issue also applies to other typical bioinformatics problems, such as other types of PTMs (such as phosphorylation [41], lysine PTMs [42], cleavage sites [43–45] etc.) and protein-protein interaction prediction [46], for which the selection of negative samples should be exercised with caution. This issue also highlights the significance of using PU-learning algorithms to address such tasks and employing only positive and unlabelled samples to train the models.

While the P1 rows are the actual probability of the glycosylation sites of the previous collection time point. For example, the P1 value of N-linked glycosylation in 2010 was 3.38%, meaning that the number of the positive samples accounts for 3.38% of the total number of samples (3.38% = (237 + 1208)/(41,526 + 1208), where 237 is the number of mislabelled non-glycosylation sites in the 2007 dataset, 1208 is the number of glycosylation sites in the 2007 dataset, and 41,526 is the total number of unlabelled sites). The P2 rows are the prior probabilities of the glycosylation sites estimated by the Elkan-Noto algorithm, while the P3 rows are the prior probabilities of the glycosylation sites estimated by the AlphaMax algorithm. In general, P3 and P2 are similar with the P1, but they are both relatively higher than P1. In addition, the value of P3 is closer to P1 than P2, which indicates the AlphaMax algorithm is more reliable than the Elkan-Noto algorithm in terms of prior probability estimation.

**Fig. 2:**
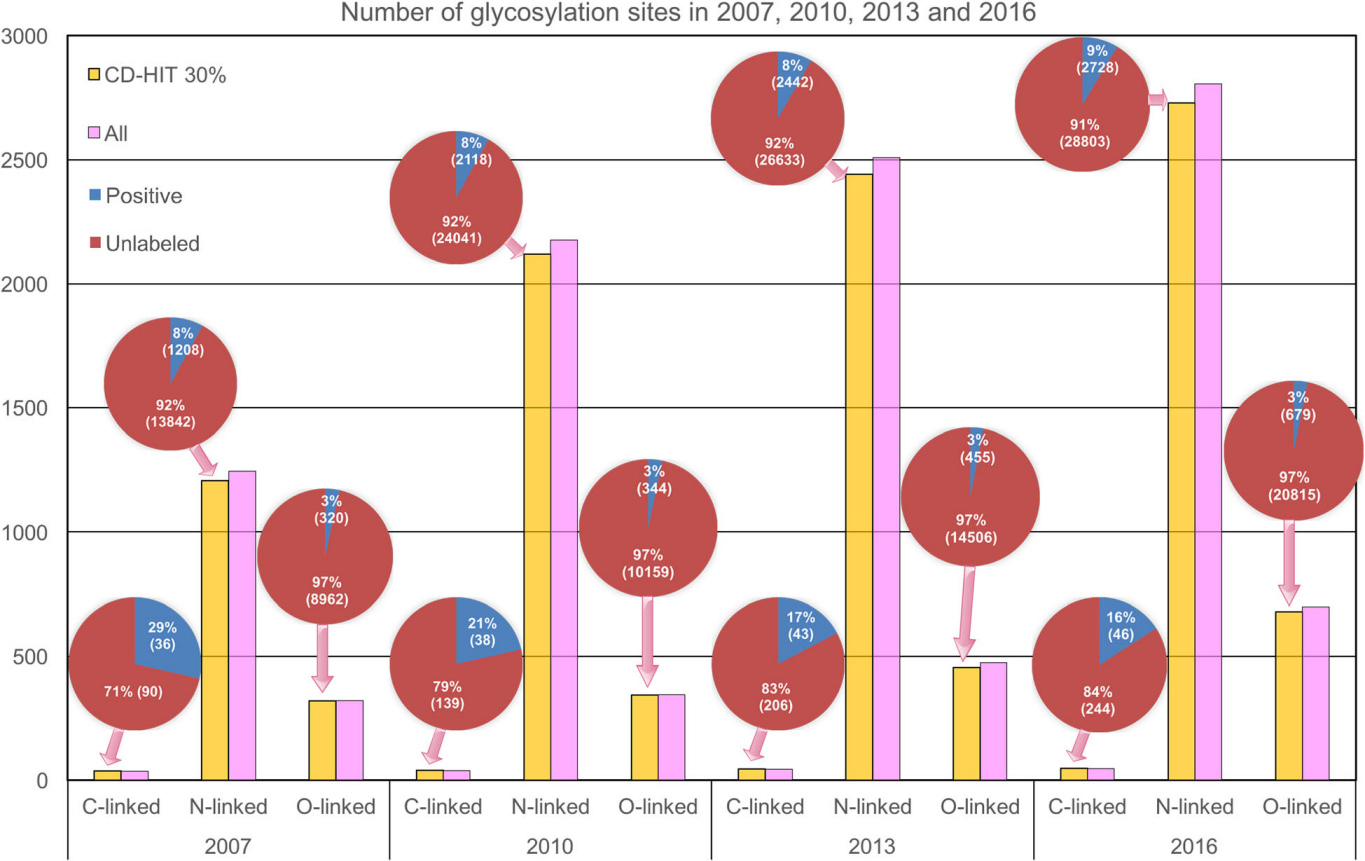
Rapid increase in the numbers of glycosylation sites and unlabelled samples in an increasing chronological order (from years 2007 to 2016)

We further analysed the proportions of unlabelled samples. Note that the unlabelled samples included non-glycosylation sites and potential glycosylation sites yet to be discovered. Based on the data shown in Table 1, we generated Fig. 2 to visually illustrate the large amounts of unlabelled samples of C-, N-, and O-linked glycosylation in chronological order from 2007 to 2016. The bar charts in Fig. 2 show the number of glycosylation sites identified each year based on Table 1, whereas the pie charts illustrate the percentage of glycosylation sites relative to unlabelled sites and associated with C-, N-, and O-linked glycosylation, respectively.

With the development of more advanced experimental technologies, increasing numbers of glycosylated proteins and corresponding glycosylation sites are being characterized (Fig. 2), with more unlabelled samples also available. For example, 1208 N-linked glycosylation sites were identified in 2007, accounting for 8% of the total number of potential N-linked glycosylation sites. This number increased to 2118 and 2442 in 2010 and 2013, respectively, whereas the percentages remained at 7%. In the case of C-linked glycosylation, a significant increase from 71 to 84% in the proportion of unlabelled samples occurred from 2007 to 2016. Altogether, these data associated with mislabelled non-glycosylation sites and the increasing number of unlabelled sites motivated us to explore the possibility of employing the PU-learning algorithms to facilitate the prediction of glycosylation sites by considering unlabelled sites.

### Performance comparison of different algorithms on the benchmark datasets

We evaluated and compared the predictive performance of the PU-learning, supervised-learning, and one-class classification algorithms on the benchmark datasets of C-, N-, and O-linked glycosylation collected from 2007, 2010, and 2013. Based on each dataset, we performed 100 randomization tests and reported the averaged performance results. For each experiment, the same numbers of unlabelled and positive samples were selected to form an initial dataset, which was further randomly split into three subsets: training set (50%), validation set (20%), and test set (30%). The validation set was used to estimate the prior probability of the positive class [36], and the training and validation sets were combined as a new training set (i.e. trainingV; Fig. 1) to retrain the classifiers. The predictive performance of the trained classifiers was evaluated using the test set, and the average performance results from 100 experiments were reported. Note that for the supervised-learning algorithms (i.e. SVM and RF), the unlabelled sites were regarded as negative samples, and we directly used the training sets to train the supervised-learning classifiers. For the one-class classification algorithms, we only used positive samples from the training sets to train the algorithms.

The average predictive performance (measured by F1, ACC, and AUC) of the classifiers on the benchmark datasets is shown in Table 3. The best F1, ACC, and AUC values for each experiment are underlined and marked in bold. These results showed that the PU-learning algorithms generally outperformed the supervised-learning and one-class classification algorithms in terms of F1, ACC, and AUC, with the only exception for the dataset of O-linked glycosylation from 2013. For the PU-learning algorithms, PA2DE, PTAN, and PNB performed best in most cases.

**Table 3:** Performance comparison of PU-learning, supervised-learning, and one-class classification algorithms on the benchmark datasets

Type	Algorithm	2007			2010			2013		
		F1	ACC	AUC	F1	ACC	AUC	F1	ACC	AUC
C	PA2DE (V2.0)	0.917 ± 0.049	0.909 ± 0.041	0.975 ± 0.061	0.917 ± 0.147	0.917 ± 0.087	0.948 ± 0.074	0.917 ± 0.034	0.923 ± 0.073	0.941 ± 0.037
	PA2DE	0.910 ± 0.051	0.904 ± 0.055	0.966 ± 0.039	0.898 ± 0.049	0.890 ± 0.054	0.925 ± 0.055	0.915 ± 0.044	0.910 ± 0.049	0.937 ± 0.046
	PAODE	0.843 ± 0.082	0.843 ± 0.077	0.912 ± 0.071	0.826 ± 0.111	0.837 ± 0.086	0.902 ± 0.083	0.870 ± 0.083	0.870 ± 0.074	0.933 ± 0.060
	PNB	0.889 ± 0.047	0.872 ± 0.060	0.943 ± 0.048	0.906 ± 0.047	0.895 ± 0.057	0.923 ± 0.047	0.908 ± 0.046	0.899 ± 0.056	0.936 ± 0.043
	PTAN	0.861 ± 0.056	0.841 ± 0.070	0.932 ± 0.049	0.887 ± 0.053	0.875 ± 0.064	0.923 ± 0.053	0.918 ± 0.046	0.912 ± 0.052	0.951 ± 0.039
	PFBC	0.869 ± 0.049	0.846 ± 0.066	0.947 ± 0.042	0.882 ± 0.045	0.866 ± 0.058	0.935 ± 0.044	0.893 ± 0.054	0.879 ± 0.068	0.940 ± 0.046
	RF^[a]^	0.847 ± 0.070	0.835 ± 0.075	0.922 ± 0.064	0.864 ± 0.058	0.856 ± 0.059	0.922 ± 0.056	0.883 ± 0.057	0.875 ± 0.062	0.941 ± 0.051
	SVM	0.810 ± 0.095	0.814 ± 0.080	0.814 ± 0.080	0.851 ± 0.083	0.853 ± 0.073	0.853 ± 0.073	0.847 ± 0.085	0.855 ± 0.068	0.855 ± 0.068
	O-SVM^[b]^	0.365 ± 0.152	0.612 ± 0.065	0.612 ± 0.065	0.400 ± 0.151	0.613 ± 0.066	0.613 ± 0.066	0.366 ± 0.136	0.606 ± 0.055	0.606 ± 0.055
	O-Classifier^[c]^	0.680 ± 0.142	0.760 ± 0.081	0.760 ± 0.081	0.662 ± 0.139	0.740 ± 0.082	0.740 ± 0.082	0.726 ± 0.122	0.785 ± 0.076	0.785 ± 0.076
N	PA2DE (V2.0)	0.933 ± 0.041	0.928 ± 0.051	0.929 ± 0.012	0.989 ± 0.011	0.985 ± 0.003	0.998 ± 0.013	0.990 ± 0.012	0.985 ± 0.003	0.998 ± 0.011
	PA2DE	0.916 ± 0.007	0.910 ± 0.008	0.914 ± 0.008	0.987 ± 0.003	0.983 ± 0.004	0.998 ± 0.002	0.988 ± 0.003	0.984 ± 0.004	0.998 ± 0.001
	PAODE	0.916 ± 0.009	0.910 ± 0.009	0.943 ± 0.026	0.940 ± 0.051	0.920 ± 0.049	0.928 ± 0.018	0.957 ± 0.004	0.938 ± 0.006	0.928 ± 0.015
	PNB	0.916 ± 0.009	0.910 ± 0.009	0.943 ± 0.026	0.948 ± 0.005	0.928 ± 0.007	0.923 ± 0.009	0.957 ± 0.004	0.938 ± 0.006	0.925 ± 0.009
	PTAN	0.985 ± 0.004	0.985 ± 0.004	0.996 ± 0.002	0.929 ± 0.010	0.899 ± 0.015	0.915 ± 0.010	0.939 ± 0.010	0.909 ± 0.015	0.920 ± 0.009
	PFBC	0.916 ± 0.008	0.910 ± 0.009	0.945 ± 0.029	0.949 ± 0.005	0.929 ± 0.007	0.937 ± 0.022	0.957 ± 0.004	0.938 ± 0.006	0.937 ± 0.021
	RF^[a]^	0.980 ± 0.005	0.980 ± 0.005	0.994 ± 0.003	0.984 ± 0.004	0.978 ± 0.005	0.997 ± 0.002	0.985 ± 0.003	0.979 ± 0.004	0.997 ± 0.002
	SVM	0.916 ± 0.007	0.910 ± 0.008	0.910 ± 0.008	0.948 ± 0.005	0.928 ± 0.007	0.912 ± 0.009	0.957 ± 0.004	0.938 ± 0.006	0.916 ± 0.009
	O-SVM^[b]^	0.551 ± 0.125	0.695 ± 0.695	0.695 ± 0.061	0.553 ± 0.108	0.585 ± 0.069	0.695 ± 0.051	0.567 ± 0.097	0.570 ± 0.066	0.701 ± 0.046
	O-Classifier^[c]^	0.868 ± 0.052	0.850 ± 0.045	0.894 ± 0.045	0.871 ± 0.037	0.855 ± 0.043	0.897 ± 0.036	0.923 ± 0.042	0.896 ± 0.051	0.904 ± 0.038
O	PA2DE (V2.0)	0.979 ± 0.007	0.979 ± 0.011	0.995 ± 0.007	0.986 ± 0.007	0.986 ± 0.010	0.997 ± 0.006	0.982 ± 0.013	0.982 ± 0.007	0.996 ± 0.012
	PA2DE	0.977 ± 0.011	0.978 ± 0.011	0.996 ± 0.012	0.983 ± 0.008	0.983 ± 0.008	0.997 ± 0.002	0.977 ± 0.013	0.977 ± 0.013	0.996 ± 0.005
	PAODE	0.968 ± 0.012	0.968 ± 0.013	0.994 ± 0.004	0.980 ± 0.010	0.980 ± 0.009	0.998 ± 0.002	0.967 ± 0.011	0.968 ± 0.010	0.995 ± 0.005
	PNB	0.984 ± 0.007	0.984 ± 0.007	0.997 ± 0.002	0.967 ± 0.010	0.966 ± 0.011	0.998 ± 0.001	0.980 ± 0.009	0.980 ± 0.008	0.997 ± 0.002
	PTAN	0.938 ± 0.016	0.936 ± 0.017	0.987 ± 0.007	0.942 ± 0.013	0.940 ± 0.014	0.988 ± 0.006	0.935 ± 0.014	0.932 ± 0.015	0.987 ± 0.006
	PFBC	0.967 ± 0.012	0.966 ± 0.013	0.998 ± 0.002	0.965 ± 0.011	0.965 ± 0.011	0.995 ± 0.003	0.963 ± 0.010	0.962 ± 0.010	0.993 ± 0.004
	RF^[a]^	0.979 ± 0.016	0.979 ± 0.016	0.994 ± 0.006	0.980 ± 0.016	0.981 ± 0.015	0.996 ± 0.004	0.967 ± 0.011	0.968 ± 0.010	0.995 ± 0.005
	SVM	0.977 ± 0.011	0.978 ± 0.011	0.996 ± 0.012	0.974 ± 0.014	0.974 ± 0.013	0.996 ± 0.005	0.981 ± 0.014	0.981 ± 0.013	0.994 ± 0.005
	O-SVM^[b]^	0.582 ± 0.053	0.691 ± 0.026	0.691 ± 0.026	0.575 ± 0.575	0.681 ± 0.028	0.681 ± 0.028	0.578 ± 0.045	0.666 ± 0.027	0.666 ± 0.027
	O-Classifier^[c]^	0.695 ± 0.039	0.593 ± 0.080	0.593 ± 0.080	0.665 ± 0.026	0.535 ± 0.059	0.535 ± 0.059	0.702 ± 0.026	0.621 ± 0.059	0.621 ± 0.059

[a] RF – Random Forest; [b] O-SVM – One-class SVM; [c] O-Classifier – One-class Classifier

### PU-learning algorithms performed best on the test datasets

To objectively compare the predictive performances, we conducted performance tests of all of the algorithms using the samples included in the dataset from 2016 rather than those from 2013, 2010, and 2007 as the positive test dataset. The numbers of tested positive samples are shown in Table 4. We only evaluated the performance of N-linked and O-linked glycosylation sites, due to the limited availability of C-linked data (only three C-linked glycosylation sites). We then randomly chose negative samples that were not labelled as glycosylation sites in all of the datasets across all the four years. This process was repeated 100 times, resulting in 100 test datasets incorporating positive datasets and different randomly selected negative datasets. We applied these 100 test datasets to evaluate the classifiers used in the benchmark test. The average predictive performance in terms of F1, ACC, and AUC are reported in Table 5.

**Table 4:** The numbers of glycosylated proteins and corresponding sites included in the test datasets

Type	Num. of Sites	Num. of Substrates
C-linked	3	2
N-linked	324	156
O-linked	244	76

**Table 5:** Performance comparison of PU-learning, supervised-learning, and one-class classification algorithms on the test datasets

Type	Algorithm	2007			2010			2013		
		F1	ACC	AUC	F1	ACC	AUC	F1	ACC	AUC
N	PA2DE (V2.0)	0.935 ± 0.003	0.934 ± 0.004	0.949 ± 0.007	0.933 ± 0.001	0.932 ± 0.002	0.950 ± 0.005	0.962 ± 0.011	0.963 ± 0.007	0.997 ± 0.008
	PA2DE	0.930 ± 0.002	0.928 ± 0.002	0.943 ± 0.004	0.930 ± 0.002	0.929 ± 0.003	0.947 ± 0.003	0.951 ± 0.018	0.952 ± 0.017	0.996 ± 0.002
	PAODE	0.929 ± 0.013	0.928 ± 0.010	0.958 ± 0.013	0.922 ± 0.051	0.923 ± 0.035	0.950 ± 0.010	0.931 ± 0.002	0.929 ± 0.003	0.950 ± 0.008
	PNB	0.929 ± 0.004	0.928 ± 0.004	0.950 ± 0.013	0.896 ± 0.074	0.887 ± 0.094	0.954 ± 0.070	0.931 ± 0.003	0.929 ± 0.003	0.955 ± 0.011
	PTAN	0.916 ± 0.013	0.913 ± 0.015	0.933 ± 0.004	0.876 ± 0.019	0.860 ± 0.024	0.938 ± 0.006	0.875 ± 0.019	0.859 ± 0.024	0.941 ± 0.003
	PFBC	0.910 ± 0.016	0.904 ± 0.018	0.939 ± 0.004	0.930 ± 0.004	0.928 ± 0.005	0.955 ± 0.012	0.893 ± 0.076	0.882 ± 0.096	0.939 ± 0.088
	RF^[a]^	0.924 ± 0.018	0.929 ± 0.016	0.994 ± 0.004	0.922 ± 0.039	0.923 ± 0.035	0.950 ± 0.010	0.931 ± 0.002	0.929 ± 0.003	0.947 ± 0.003
	SVM	0.919 ± 0.002	0.907 ± 0.002	0.935 ± 0.002	0.904 ± 0.002	0.897 ± 0.003	0.929 ± 0.003	0.931 ± 0.002	0.929 ± 0.003	0.929 ± 0.003
	O-SVM^[b]^	0.683 ± 0.009	0.740 ± 0.011	0.740 ± 0.011	0.689 ± 0.010	0.748 ± 0.012	0.748 ± 0.012	0.689 ± 0.010	0.747 ± 0.012	0.747 ± 0.012
	O-Classifier^[c]^	0.820 ± 0.032	0.847 ± 0.023	0.847 ± 0.023	0.849 ± 0.029	0.865 ± 0.023	0.865 ± 0.023	0.836 ± 0.029	0.857 ± 0.021	0.857 ± 0.021
O	PA2DE (V2.0)	0.933 ± 0.046	0.930 ± 0.053	0.986 ± 0.010	0.945 ± 0.021	0.943 ± 0.014	0.995 ± 0.012	0.986 ± 0.013	0.986 ± 0.020	0.997 ± 0.031
	PA2DE	0.928 ± 0.052	0.924 ± 0.060	0.978 ± 0.022	0.932 ± 0.018	0.928 ± 0.050	0.981 ± 0.019	0.974 ± 0.019	0.974 ± 0.019	0.994 ± 0.006
	PAODE	0.848 ± 0.061	0.816 ± 0.090	0.976 ± 0.006	0.923 ± 0.017	0.926 ± 0.014	0.984 ± 0.019	0.952 ± 0.015	0.955 ± 0.013	0.996 ± 0.007
	PNB	0.906 ± 0.030	0.896 ± 0.036	0.989 ± 0.002	0.926 ± 0.017	0.921 ± 0.020	0.991 ± 0.002	0.970 ± 0.012	0.969 ± 0.012	0.997 ± 0.001
	PTAN	0.798 ± 0.075	0.832 ± 0.051	0.961 ± 0.011	0.844 ± 0.044	0.815 ± 0.067	0.924 ± 0.052	0.886 ± 0.064	0.867 ± 0.090	0.972 ± 0.035
	PFBC	0.838 ± 0.046	0.810 ± 0.070	0.916 ± 0.057	0.910 ± 0.031	0.901 ± 0.038	0.990 ± 0.002	0.904 ± 0.073	0.886 ± 0.103	0.991 ± 0.004
	RF^[a]^	0.914 ± 0.019	0.919 ± 0.016	0.984 ± 0.015	0.923 ± 0.017	0.926 ± 0.014	0.984 ± 0.019	0.952 ± 0.015	0.955 ± 0.013	0.996 ± 0.007
	SVM	0.924 ± 0.014	0.919 ± 0.016	0.988 ± 0.002	0.930 ± 0.008	0.975 ± 0.009	0.975 ± 0.009	0.920 ± 0.019	0.924 ± 0.020	0.974 ± 0.001
	O-SVM^[b]^	0.677 ± 0.016	0.537 ± 0.033	0.537 ± 0.033	0.661 ± 0.006	0.506 ± 0.012	0.506 ± 0.012	0.665 ± 0.007	0.007 ± 0.016	0.506 ± 0.016
	O-Classifier^[c]^	0.141 ± 0.141	0.529 ± 0.033	0.529 ± 0.033	0.135 ± 0.100	0.532 ± 0.025	0.532 ± 0.025	0.144 ± 0.116	0.527 ± 0.035	0.527 ± 0.035

[a] RF – Random Forest; [b] O-SVM – One-class SVM; [c] O-Classifier – One-class Classifier

The predictive performance of the algorithms on the test datasets showed that PA2DE (V2.0) performed best for both N- and O-linked glycosylation site prediction in terms of F1 and ACC. Additionally, PNB achieved the highest AUC values on the O-linked datasets of 2007 and 2013. On the N-linked dataset of 2010, PFBC achieved the best AUC value, while RF achieved the best AUC value when trained using the 2007 N-linked glycosylation datasets, while SVM achieved the best ACC value on the O-linked dataset of 2010. Compared to the PU-learning and supervised-learning algorithms, the one-class learners performed the worst across all these years in terms of AUC and accuracy.

**Fig. 3:**
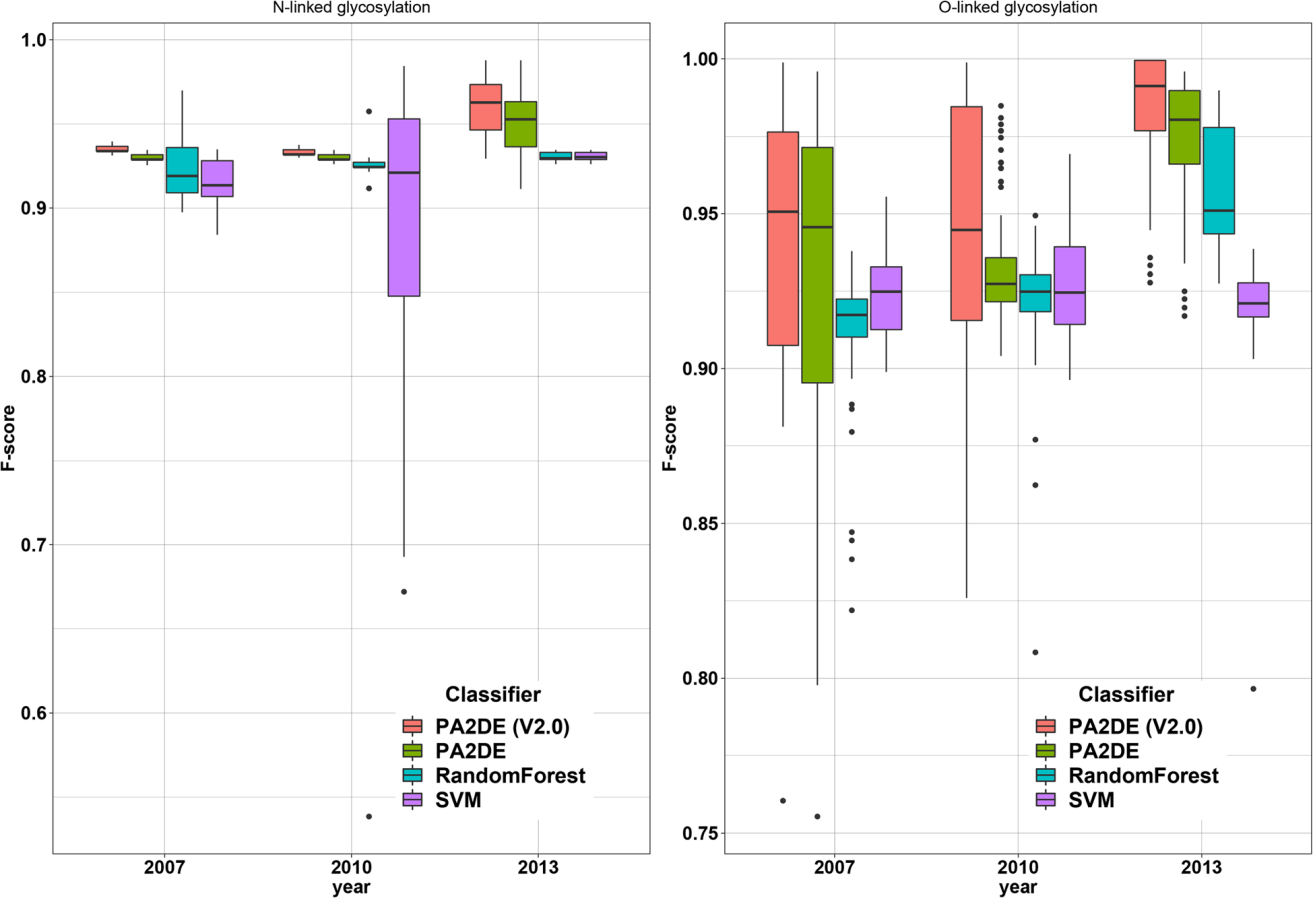
Boxplots showing that PA2DE outperformed the RF and SVM algorithms in terms of F1 score on the test datasets

As shown in Table 5, in most cases, PA2DE (V2.0) performed best among the PU-learning algorithms on the test datasets. To examine the statistical significance of F1 improvement by PA2DE (V2.0), we performed a Student’s *t*-test to compare the results from PA2DE (V2.0), PA2DE, RF, and SVM. Table 6 provides the calculated *p*-values, which indicate that the F1 of PA2DE was significantly (*p* ≤ 0.01) higher than that for RF and SVM according to eight pairwise tests (marked in bold) among a total of 12 tests. Figure 3 plots the distributions of F1 scores for these algorithms on the test datasets, with the average F1 scores for PA2DE substantially higher than that for RF and SVM.

**Table 6:** Statistical significance of PA2DE performance in terms of F1 scores relative to the RF and SVM algorithms on the test datasets

Type	Algorithm	2007	2010	2013
N-linked	PA2DE	6.35E-04	0.0369	6.07E-23
	Random Forest			
	PA2DE	5.61E-21	8.10E-06	6.96E-23
	SVM			
	PA2DE (V2.0)	2.44E-09	0.0233	7.35E-40
	Random Forest			
	PA2DE (V2.0)	1.09E-32	1.34E-06	8.33E-40
	SVM			
O-linked	PA2DE	0.0104	6.10E-04	1.86E-09
	Random Forest			
	PA2DE	0.4566	0.0210	9.64E-16
	SVM			
	PA2DE (V2.0)	6.23E-04	1.04E-04	6.53E-20
	Random Forest			
	PA2DE (V2.0)	0.0986	9.19E-04	8.19E-29
	SVM

### Comparison with existing methods and web server implementation

Thus far, we have used four time-scaling datasets collected from the UniProt database to compare the prediction performance of PA2DE (V2.0) with PU-learning, supervised learning and one-class classification algorithms. The results demonstrated that the PA2DE (V2.0) algorithm achieved the best performance in most scenarios.

In this section, we constructed a complete dataset with experimentally validated human glycosylation sites collected from the UniProt, dbPTM [47], and PhosphoSitePlus [48] databases to make the performance comparison with the existing methods. For the data extracted from the UniProt database, we only considered glycosylation sites with ECO code ECO:0000269, which indicates the manually curated information with published experimentally evidence (https://www.uniprot.org/help/evidences). We further implemented an online web server using an optimized PA2DE (V2.0) algorithm trained on this new dataset. According to a previous study [28], N-linked glycosylation is generally associated with a N[!P][ST][!P] motif which is highly specific and aids in the predictor learning. Thus, we further classified the N-linked glycosylation sites into two subsets: a motif subset which contained all the glycosylation sites located in such motif and a non-motif set which did not include any obvious motifs. In addition, given that the O-linked glycosylation usually occurs on two different types of residues Serine (S) and Threonine (T), we constructed two different models for each residue type separately. We then removed the redundant sequences from this dataset with the sequence identity of 30% by using the CD-HIT program. The statistical summary of this dataset is shown in Table 7.

**Table 7:** A statistical summary of glycosylated proteins and glycosylation sites collected from UniProt, dbPTM and PhosphoSitePlus

Type	Before redundancy removal		After redundancy removal	
	Num. of Proteins	Num. of Sites	Num. of Proteins	Num. of Sites
C-linked	13	134	10	109
N-linked (motif)	1103	3850	770	2669
N-linked (non-motif)	100	158	91	146
O-linked (S)	192	683	165	602
O-linked (T)	169	2150	155	2095

We randomly split the dataset into the training sets and independent test sets with the ratio of 7 to 3. The training sets were used for constructing the PA2DE (V2.0) model for the web server and the independent test sets were used for benchmarking the predictive performance with other existing methods. A statistical summary of the training set and independent test set is shown in Table 8.

**Table 8:** Numbers of glycosylation sites included in the training sets and independent test sets

Type	Training set	Independent test set
C-linked	76	33
N-linked (motif)	1869	800
N-linked (non-motif)	102	44
O-linked (S)	421	181
O-linked (T)	1467	628

We adopted the PU-learning protocol suggested in a recent work, MutPred2 [35] to re-train the PA2DE (V2.0) model based on the top 100 ranked features of the training set. The unlabelled dataset was first generated by randomly selecting 20 non-glycosylation sites from each glycosylated protein in the training data set. Then, the positive and unlabelled datasets were used to perform feature selection. The mRMR algorithm was employed to identify the top 100 ranked features for each type of glycosylation. The feature selection results are shown in Table 9.

**Table 9:** The number of different selected feature groups as result of feature selection

Type	AAC	Auto-correlation	CTD	Sequence-order	Pseudo-AAC	AAindex
C-linked	3	23	2	4	5	63
N-linked (motif)	2	1	4	2	4	87
N-linked (non-motif)	4	1	3	2	4	86
O-linked (S)	11	7	13	3	3	63
O-linked (T)	8	4	8	2	2	76

For each type of glycosylation, a final unlabelled set was generated by further randomly selecting 10,000 non-glycosylation sites from the training set. In doing so, we ensure the estimation of class prior probability is fairly low (1 × 10^− 4^). As the glycosylation site prediction is a problem with class imbalance, the mislabelled samples exist in the unlabelled set with a relatively low fraction. For these types of glycosylation with fewer than 10,000 unlabelled samples, all the non-glycosylation sites were included. Then, a five-fold cross-validation test was performed on the training datasets. The summary of the training datasets for each type of glycosylation and the corresponding predictive performance are shown in Table 10.

**Table 10:** Summary of the training datasets and performance results of PA2DE (V2.0)

Type	Number of Sites		AUC	ACC	F1
	Positive	Unlabelled			
C-linked	76	258	0.997	0.981	0.981
N-linked (motif)	1869	7111	0.927	0.886	0.894
N-linked (non-motif)	102	4888	0.874	0.974	0.973
O-linked (S)	421	8331	0.974	0.972	0.972
O-linked (T)	1467	10,000	0.876	0.857	0.859

**Fig. 4:**
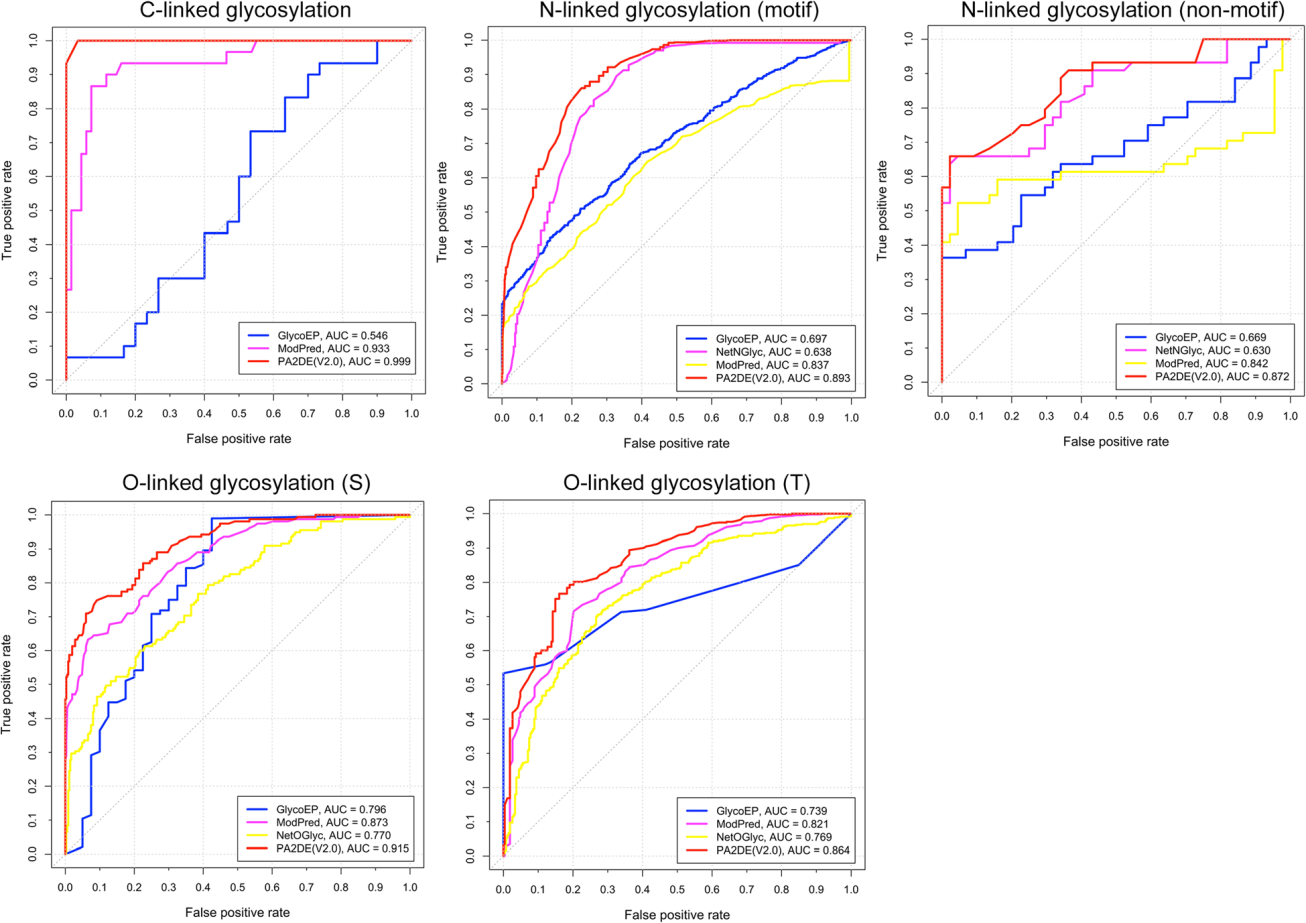
ROC curves for PA2DE (V2.0), NetNGlyc, NetOGlyc, GlycoEP, and ModPred on independent test datasets

In order to objectively evaluate the performance of our method, we compared the predictive performance of PA2DE (V2.0) with several state-of-the-art methods, including GlycoEP, NetNGlyc, NetOGlyc, and ModPred, on the independent test datasets. In order to perform the prediction, the protein sequences of the independent test datasets were submitted to the web servers/softwares of these methods with the default or recommended settings to obtain the prediction results, which were then used for evaluating the predictive performance of these methods. We randomly selected the equal number of non-glycosylation sites to the number of glycosylation sites as the negative samples from the glycosylated proteins in the independent test datasets. For example, if a protein sequence contained *n* N-linked glycosylation sites, we randomly selected *n* amino acids (N) that were not labelled as N-linked glycosylation sites as the negative samples. The predictive performance for different types of glycosylation sites are shown in Table 11. We also generated the ROC curves (shown in Fig. 4) to evaluate and compare the performance of different methods.

**Table 11:** Performance comparison results between different methods on the independent test datasets

Type	Methods	AUC	ACC	F1
C-linked	PA2DE (V2.0)	0.999	0.983	0.984
	GlycoEP	0.546	0.600	0.647
	ModPred	0.933	0.933	0.938
N-linked (motif)	PA2DE (V2.0)	0.893	0.815	0.820
	GlycoEP	0.697	0.637	0.638
	NetNGlyc	0.638	0.616	0.627
	ModPred	0.837	0.782	0.791
N-linked (non-motif)	PA2DE (V2.0)	0.872	0.761	0.758
	GlycoEP	0.669	0.648	0.644
	NetNGlyc	0.630	0.716	0.675
	ModPred	0.842	0.807	0.773
O-linked (S)	PA2DE (V2.0)	0.915	0.859	0.766
	GlycoEP	0.796	0.787	0.848
	ModPred	0.873	0.770	0.670
	NetOGlyc	0.770	0.662	0.583
O-linked (T)	PA2DE (V2.0)	0.864	0.793	0.827
	GlycoEP	0.739	0.694	0.747
	ModPred	0.821	0.746	0.781
	NetOGlyc	0.769	0.718	0.763

As shown in Table 11 and Fig. 4, the performance comparison results indicate that for all five types of glycosylation, PA2DE (V2.0) achieved the best performance in terms of AUC. While for N-linked (non-motif) glycosylation, ModPred achieved the best ACC (0.807) and F1 score (0.773), while for O-linked (S) glycosylation, GlycoEP achieved the best F1 score (0.848).

Based on the trained models of PA2DE (V2.0), we further implemented an online web server that enables the users to predict potential novel glycosylation sites. The web server is freely available at http://glycomine.erc.monash.edu/Lab/GlycoMine_PU/, developed using Java Server Pages and managed by Tomcat 7 on a Linux server.

## Conclusions

In this study, we have proposed a new computational method, PA2DE (V2.0), to address the task of protein glycosylation site prediction in the PU-learning scenario. A variety of algorithms, including supervised-learning (SVM and RF), PU-learning (PA2DE, PAODE, PNB, PTAN, and PFBC), and one-class classification algorithms (OneClassClassifiers and one-class SVMs) were extensively benchmarked, evaluated and compared with our proposed method in this study. Both benchmarking and independent tests showed that our proposed method achieved a competitive predictive performance compared with several supervised-learning algorithms for glycosylation-site prediction. Performance comparison results with the other existing methods indicate that the proposed method is capable of accurately predicting protein glycosylation sites. A publicly available web server has been implemented to facilitate the prediction of potential glycosylated protein substrates and glycosylation sites. With the success of applying PU-learning scheme to protein glycosylation prediction in this study, we anticipate that such methods can be widely employed to facilitate the prediction of other protein functional sites, including other different types of PTMs.

## Methods

### Data collection and pre-processing

As noted, four datasets comprising experimentally verified human C-, N-, and O-linked glycosylation sites from years 2007, 2010, 2013, and 2016 were collected from the UniProt database. To avoid overfitting and performance over-estimation, we removed sequence redundancy from all four datasets using the CD-HIT program [49] by setting the identity between any two sequences to ≤30%.

The predictive performance of traditional supervised-learning algorithms is contingent on the quality of both positive and negative samples. Similar to previous studies [29, 30], experimentally determined glycosylation sites were used as positive samples (Table 2). An increasing number of C-, N-, and O-linked glycosylation sites and glycosylated proteins were identified from 2007 to 2016 (e.g., increasing from 1245 N-linked glycosylation sites in 2007 to 2805 in 2016). In-depth analysis of annotation changes in the data spanning these 4 years is provided in the section “Dataset analysis”. Importantly, this indicated that a sizable number of non-glycosylation sites previously mislabelled and treated as negative sites (due to limitations in the experimental methods at the time) should be used as valid positive sites. Obviously, the inclusion of such mislabelled data will affect the performance evaluation of glycosylation-prediction models. Therefore, it is reasonable to assume that a portion of other experimentally unexplored residues, including tryptophan, asparagine, serine, and threonine, can be potentially identified as C-, N-, or O-linked glycosylation sites as experimental technologies continue to advance. Note that all current computational methods for glycosylation prediction were developed based on the labelling of positive and negative samples, which is consistent with traditional supervised-learning schemes. Therefore, it is difficult for existing methods to retrain or update the models in order to keep pace with rapidly updated data, especially concerning previously mislabelled negative samples. In this study, we predicted glycosylation sites by using a PU-learning scheme.

For benchmarking tests, we employed glycosylation sites retrieved from 2007, 2010, and 2013 as the positive samples used to train the classifiers. For traditional supervised-learning models (i.e., SVMs and RF), we randomly selected the same number of non-glycosylation sites as negative samples in order to construct the negative training datasets. As noted, such negative samples could be mislabelled due to limitations in experimental technologies. By contrast, for PU-learning models, such negative samples were treated as unlabelled samples. Because one-class learners only require information concerning the target class (i.e., glycosylation sites), there is no need to assign any negative or unlabelled samples for such models.

For the performance test set, we selected glycosylation sites that were experimentally annotated exclusively in 2016 as positive test samples. Negative samples (i.e., those having been consistently labelled as non-glycosylation sites across all 4 years) were randomly selected to constitute the negative dataset, with an equal number of positive samples used for each type of glycosylation. This random-sampling procedure was repeated 100 times. Due to insufficient test data for C-linked glycosylation (only three sites available), the performance test was constructed only for N- and O-linked glycosylation.

### Feature extraction and selection

A local sliding window comprising 15 residues (i.e., seven upstream residues and seven downstream residues centred on the glycosylation site) [29] was used for feature extraction. This 15-residue peptide can be represented as [50]:

1 $$ \boldsymbol{P}={p}_1{p}_2\dots {p}_8\dots {p}_{14}{p}_{15}, $$

where *p*_*i*_ denotes the *i*-th residue of the peptide, ***P,*** and *p*_8_ denotes the glycosylation site. In this study, we extracted six groups of sequence-derived features to encode a peptide and train the machine-learning model. The first group consists of: 1) 20 amino acid compositions [51], and 2) 400 dipeptide amino acid compositions [52].

The second group includes three different types of autocorrelation features: 1) 240 normalized Moreau-Broto autocorrelation features [53, 54]; 2) 240 Moran autocorrelation features [55]; and 3) 240 Geary autocorrelation features [56]. The autocorrelation features measure the level of correlation between two peptide sequences according to their physicochemical properties.

The third group is ‘Composition-Transition-Distribution (CTD)’ [57], which includes three types of features: 1) 21 composition features, 2) 21 transition features, and 3) 105 distribution features. These features are calculated based on physicochemical properties that represent the amino acid-specific distribution of a specific structural or physicochemical property within a peptide.

The fourth group includes two sequence-order-feature sets: 1) 60 sequence-order-coupling number features, and 2) 100 quasi-sequence-order features [58].

The fifth group contains two types of pseudo-amino-acid-composition features: 1) 50 type I features; and 2) 50 type II features [52].

The sixth group contains 8400 AAindex features extracted from the AAindex database [59].

A total of 9927 features were extracted and calculated. It is possible that such a high-dimensional feature set might contain certain noisy and irrelevant features, resulting in disfavorable model training and decreased predictive performance. To remove such features, we applied the mRMR (minimum Redundancy and Maximum Relevance) algorithm [40] and selected the top 100 features contributing the most to each C-, O-, and N-linked glycosylation event. mRMR evaluates the relevance and redundancy of two features, *x* and *y*, based on mutual information, which is defined as:

2 $$ I\left(x,y\right)=\iint p\left(x,y\right)\log \frac{p\left(x,y\right)}{p(x)p(y)} dxdy, $$

where *p*(*x*, *y*) is the joint probability of feature *x* and *y*, where *p*(*x*) and *p*(*y*) are marginal probabilities.

### PU-learning algorithms

Current PU-learning algorithms can be generally categorized into two main types. The first type has been implemented as a ‘*two-step*’ strategy, where the algorithms identify reliable negative samples from the unlabelled dataset first and then employ both the positive samples and these identified reliable negative samples to train a classifier in the second step. This procedure needs to be repeated until a certain threshold (e.g., a performance measure, such as Matthews’s correlation coefficient or AUC) is achieved. To the best of our knowledge, this represents a predominant strategy currently practiced in bioinformatics research and has been adopted for identification and prediction of disease-associated genes from the human genome [60–62], protein pupylation prediction [63, 64], kinase substrates identification [65], protein subcellular localization prediction [66], and drug interactions prediction [67].

The second type focuses on evolving traditional supervised-learning algorithms to enable learning from both positive and unlabelled data. To date, several promising algorithms have been reported based on the evolution of classic supervised-learning algorithms, including decision trees (C4.5) [68] and Bayesian classifiers [69]. For example, POSC4.5 [70] was proposed based on the C4.5 algorithm, and based on Bayesian theory, He et al. [71] proposed a series of Bayesian classifiers for PU-learning, including PTAN (Positive Tree Augmented Naïve Bayes), PFBC (Positive Full Bayesian Network Classifier), PNB (Positive Naïve Bayes), and PAODE (Positive Averaged One-Dependence Estimators), according to the ‘*selected completely at random*’ assumption [36]. Previously, we proposed PA*n*DE (Positive Averaged *n*-Dependence Estimators) [72], which extends the A*n*DE algorithm [73] based on the ‘*selected completely at random*’ assumption.

A*n*DE relaxes the attribute-independence assumption by selecting *n* parent-attributes and assuming that all other attributes are independent of the given class label. The classification algorithm used by PA*n*DE for a sample, **x**, is described as follows:

3 $$ \mathrm{PA}n\mathrm{DE}\left(\mathbf{x}\right)=\arg \underset{y}{\max}\sum \limits_{S\in \left(\begin{array}{c}A\\ {}n\end{array}\right)}\delta \left({x}_S\right)P\left(y,{x}_S\right)\prod \limits_{m=1}^nP\left({x}_m|y,{x}_S\right), $$

where *x*_*m*_ denotes the value of attribute *X*_*m*_, $$ \left(\begin{array}{c}A\\ {}n\end{array}\right) $$denotes the set of all size-*n* subsets of the attribute set *A* = {1,…,*k*}, and *x*_*S*_ denotes a tuple of parent attributes having *n* attributes. The *δ*(*x*_*S*_) function is used to avoid using parent attributes, the values of which do not occur in the training data, and *δ*(*x*_*S*_) = 1 if *x*_*S*_ occurs in the training dataset [otherwise *δ*(*x*_*S*_) = 0]. Note that in the case that all *δ*(*x*_*S*_) = 0, eq. (1) becomes:

4 $$ \mathrm{PA}n\mathrm{DE}\left(\mathbf{x}\right)=\mathrm{PA}\left(n-1\right)\mathrm{DE}\left(\mathbf{x}\right). $$

Empirical studies showed that PA*n*DE outperformed PNB and PAODE according to evaluation using 20 UCI datasets and the protein glycosylation datasets collected in GlycoMine [72].

The original PA*n*DE algorithm applies the estimation method based on the ‘*selected completely at random*’ assumption to estimate the class priors, which has been shown to overestimate class priors, especially in cases where the true class priors are extremely small [74, 75]. Recently, a very useful algorithm, termed AlphaMax [35, 74, 75], has been proposed to provide a new solution to estimate class priors. Considering that glycosylation prediction is a class imbalance problem and the mislabelled data in unlabelled set is low fraction, we thus used this new estimation method for class prior estimation in the PA*n*DE algorithm, referred to as PA*n*DE (V2.0).

Based on Bayes’ theorem and the conditional independence assumption, PNB was initially devised based on a multinomial model of naïve Bayes (NB) for text classification [76]. This algorithm requires users to provide the prior probability of a positive class in order to estimate the probability for each class. Further, He et al. [71] extended PNB based on the ‘*selected completely at random*’ assumption in order to handle general classification tasks, with no requirement to provide prior probability.

PAODE [36] was proposed based on the AODE [77] algorithm (i.e., A1DE), which is a special version of A*n*DE (*n* = 1) that relaxes the attribute independence assumption by using one super-parent attribute and considering all other attributes as conditionally independent, given this super-parent. Similarly, the proposed PAODE algorithm (i.e., PA1DE) is a special version of PA*n*DE, where *n* = 1.

PTAN is a another version of the tree-augmented NB (TAN) [78] algorithm for positive-unlabeled learning. The TAN algorithm approximates interactions between attributes by using a tree structure imposed upon the NB structure. TAN-structure learning occurs through computation of the conditional mutual information between two attributes, given a specific class label.

PFBC was proposed based on full Bayesian network classifier (FBC) [79], where the conditional probability table for each attribute is a decision tree. Learning an order of attributes is the most important process in constructing a full Bayesian network. The experimental results reported by He et al. [71] demonstrate that PFBC is more robust against unlabelled data.

In this study, we compared the predictive performance of our newly proposed PA2DE (V2.0) algorithm with the other five Bayesian PU-learning algorithms PA2DE, PNB, PAODE, PTAN, and PFBC for predicting glycosylation and further compared its predictive performance with several supervised-learning and one-class classification algorithms.

### Supervised-learning algorithms

Two representative supervised-learning algorithms, RF and SVMs were used to compare the predictive performance of PU-learning and one-class classification algorithms. These two algorithms have been widely used to solve a variety of bioinformatics tasks and also in protein glycosylation prediction [11, 29, 30, 80–83] with the results providing outstanding predictive performance. In our study, we employed implementations of RF and SVM based on the WEKA machine-learning platform [84]. All corresponding parameters used for the two algorithms were set as the default values.

### One-class classification algorithms

One-class classification algorithms identify samples of a specific class by learning from a training set containing samples only from this class. One-class classification has been widely applied in a variety of real-world scenarios, such as outlier [85] and novelty detection [86]. We also attempted to apply this learning method to glycosylation identification. We selected two state-of-the-art one-class classification algorithms implemented in WEKA (one-class SVMs [87] and OneClassClassifiers [88]) and used the same positive samples to train these one-class classifiers. A testing sample was predicted as ‘1’ if the trained classifiers regarded the sample as positive; otherwise, it was predicted as ‘?’. We regarded samples predicted as ‘?’ as predicted negative samples. Based on this strategy, we evaluated the performance of the one-class classifiers using the same performance measures as those for the supervised- and PU-learning methods.

### Performance evaluation

Three performance measures were employed to evaluate the predictive performance of the supervised- and PU-learning schemes and facilitate comparisons between different methods, including AUC, F1, [36, 70] and ACC (Accuracy). These measurements are defined as follows:

5 $$ \mathrm{F}1=\frac{2\times Precision\times Recall}{Precision+ Recall}, $$

6 $$ \mathrm{ACC}=\frac{TP+ TN}{TP+ TN+ FP+ FN}, $$

where *Precision* and *Recall* in (5) are respectively defined as

7 $$ \mathrm{Precision}=\frac{TP}{TP+ FP}, $$

8 $$ \mathrm{Recall}=\frac{TP}{TP+ FN}. $$

where *TP*, *TN*, *FP*, and *FN* represent the numbers of true positives, true negatives, false positives, and false negatives, respectively.

## Abbreviations

ACC: Accuracy
AUC: Area Under the ROC Curve
FN: False Negative
FP: False Positive
PA*n*DE: Positive Averaged *n*-Dependence Estimators
PAODE: Positive Averaged One-Dependence Estimators
PFBC: Positive Full Bayesian Network Classifier
PNB: Positive Naïve Bayes
PTAN: Positive Tree Augmented Naïve Bayes
PTM: Post-Translational Modification
PU-learning: Positive-Unlabelled learning
RF: Random Forest
SVM: Support Vector Machine
TN: True Negative
TP: True Positive
